# Design and Fabrication of a Dual-Axis MEMS Electrostatic Micromirror Based on a Planar Comb Drive

**DOI:** 10.3390/mi17030278

**Published:** 2026-02-24

**Authors:** Mumu Li, Wenlong Jiao, Kun Huang, Botao Wang, Zhihua Dai, Yang Gao, Huiliang Cao, Huikai Xie

**Affiliations:** 1 State Key Laboratory of Extreme Environment Optoelectronic Dynamic Measurement Technology and Instrument, North University of China, Taiyuan 030051, China; 2 School of Instrument and Electronics, North University of China, Taiyuan 030051, China; 3 Bit Chongqing Institute of Microelectronics and Microsystems, Chongqing 400000, China; 4 School of Automation, Beijing Institute of Technology, Beijing 100081, China; 5 Quanzhou Yunjian Measurement Control and Perception Technology Innovation Research Institute, Quanzhou 362000, China; 6 School of Computer Engineering, Nanjing Institute of Technology, Nanjing 211167, China; 7 School of Integrated Circuits and Electronics, Beijing Institute of Technology, Beijing 100081, China

**Keywords:** electrostatic drive, planar comb drive structure, dual-axis torsion, resonant operating mode, Lissajous scan patterns

## Abstract

This paper designs and fabricates an electrostatic-driven dual-axis MEMS micromirror capable of out-of-plane torsional motion about both the X and Y axes. Both torsional axes employ planar comb structures for their drive mechanisms, effectively reducing the fabrication complexity. By leveraging the structural asymmetry introduced during processing in conjunction with resonant operating modes, the inherent disadvantage of planar comb structures for torsional motion is overcome. This study explores the operating principle, structural design, performance simulation, fabrication process, and testing of the micromirror. It proposes an indirect simulation method suitable for planar comb drive structures, providing theoretical support for device fabrication. During fabrication, optimising the removal of isolation material through oxygen–silicon growth enhances the reliability of subsequent processes. Test results demonstrate that the fabricated MEMS micromirror achieves a 26°×22° field of view at a 35 V drive voltage, outputting Lissajous-type scanning patterns. This design aims to propose an indirect simulation method and optimise the process accordingly. Experimental test results show that the simulation method is relatively accurate, with minimal deviation from actual tests. Process optimization improves wafer cleanliness and reduces the time cost of the corresponding process.

## 1. Introduction

MEMS scanning mirrors are micro-actuators integrating micro-mirrors, micro-elastic beams, and micro-drivers, enabling the manipulation and modulation of light for sensing, communication, and display applications [1]. Compared to conventional twisting mirrors, MEMS scanning mirrors offer advantages such as rapid response, low power consumption, compact size, and cost-effectiveness due to their MEMS fabrication process. Consequently, they have found extensive application in fields including Lidar [2,3,4], laser projectors [5,6,7], optical coherence tomography (OCT) [8,9,10,11], and biomedical imaging [12,13].

MEMS micromirrors can be configured in multiple combinations based on their drive principles, torsional dimensions, and operating modes to suit diverse applications. Specifically, drive principles are broadly categorised into four types: electrostatic drive [14], electromagnetic drive [15], electrothermal drive [16], and piezoelectric drive [17]. Mirror torsional dimensions include one-dimensional (single-axis torsion) [18], two-dimensional (dual-axis torsion) [19], and three-dimensional (dual-axis torsion with vertical motion). Operating modes comprise quasi-static and resonant modes. As scanning constitutes the primary function of MEMS micromirrors, two-dimensional designs are most prevalent. Different scanning effects are achievable when the two torsion axes operate in distinct modes: dual-linear scanning occurs when both axes function quasi-statically, while Lissajous scanning is achieved when both axes operate in resonant mode [20,21,22]. When one axis operates in quasi-static mode and the other in resonant mode, grating scanning occurs [23,24]. This paper primarily focuses on electrostatic micromirrors operating in dual-axis resonant mode.

Extensive research has been conducted both domestically and internationally on electrostatic-driven two-dimensional micromirrors. Researchers including He Siyuan from Canada have reported a two-dimensional electrostatically driven scanning mirror. This actuator employs an electrostatic repulsion force, enabling a larger scanning angle. The principle of electrostatic repulsion is achieved by designing a specialized comb structure that causes the electrostatic attractive force to manifest as a repulsive force. The final fabricated micromirror can achieve a mechanical torsional angle of 1.5° in both the fast axis and the slow axis [25]. The Fraunhofer Institute in Germany reported a two-dimensional electrostatic scanning mirror that utilizes a vertical comb drive actuator, with the mirror being supported by three torsional beams. Featuring a mirror diameter of 7 mm, it achieves two-dimensional resonant motion at a resonant frequency of approximately 1.5 kHz. Both the fast axis and the slow axis can attain a torsional angle of ±6.25° [26]. Liu Yaobo and Qiao Dayong et al. from Northwestern Polytechnical University reported a two-dimensional electrostatic scanning mirror employing planar comb drivers for both axes, achieving scanning angles of ±7.28° and ±5.36° for the fast and slow axes, respectively [27]. The process of filling isolation trenches with polysilicon is relatively complex, and the filling effect is difficult to control. By placing two one-dimensional micromirrors perpendicularly, two-dimensional scanning can also be achieved. Gloria Silva et al. [28] proposed a single-axis rotating micro-mirror based on a vertical comb drive. This enables laser displacement along two perpendicular directions through the coordination of two single-axis MEMS micro-mirrors, thereby obtaining a raster scanning scheme. In summary, two-dimensional micromirrors generally employ vertical comb drive configurations to enhance their twist angles, which significantly increases fabrication complexity. Polysilicon is predominantly used for trench isolation filling, but yields suboptimal results. Although alternative polymer BCB filling schemes exist, direct BCB deposition necessitates time-consuming and labour-intensive removal processes.

On this basis, the present paper introduces a dual-axis MEMS micromirror driven by electrostatic forces and employing a planar comb structure for actuation. An indirect simulation approach is proposed to model the torsional behaviour of the planar comb drive structure, providing theoretical support for device fabrication. Concurrently, the process for removing the BCB layer is optimised to enhance the reliability of subsequent manufacturing steps. Section 2 outlines the operating principles and key parameters of the micromirror; Section 3 details the structural design and simulates its relevant performance characteristics; Section 4 describes the fabrication process and presents test results; and Section 5 summarises the work undertaken in this paper.

## 2. Micromirror Operating Principles and Key Parameters

### 2.1. Micromirror Operating Principles

This section explains the operating principles of MEMS micromirrors based on their drive mechanisms. The primary drive methods include electrostatic drive, electromagnetic drive, piezoelectric drive, and electrothermal drive. Electrostatic drive is the most prevalent method, utilising electrostatic forces to deflect the mirror. Electromagnetic drive is suited for applications requiring large deflection angles and high torque, employing the Ampère force and Lorentz force to achieve mirror deflection. Piezoelectric drive harnesses the inverse piezoelectric effect of piezoelectric materials to induce mirror deflection. Finally, electrothermal drive leverages thermal expansion effects to cause mirror deflection. Table 1 summarises the characteristics of these four drive methods. Comparative analysis reveals that electrostatic drive offers low power consumption, rapid response times, straightforward fabrication processes, and high compatibility with integrated circuits. Consequently, to minimise overall manufacturing complexity for the micromirror, the electrostatic drive method has been adopted for this project.

**Figure 1:**
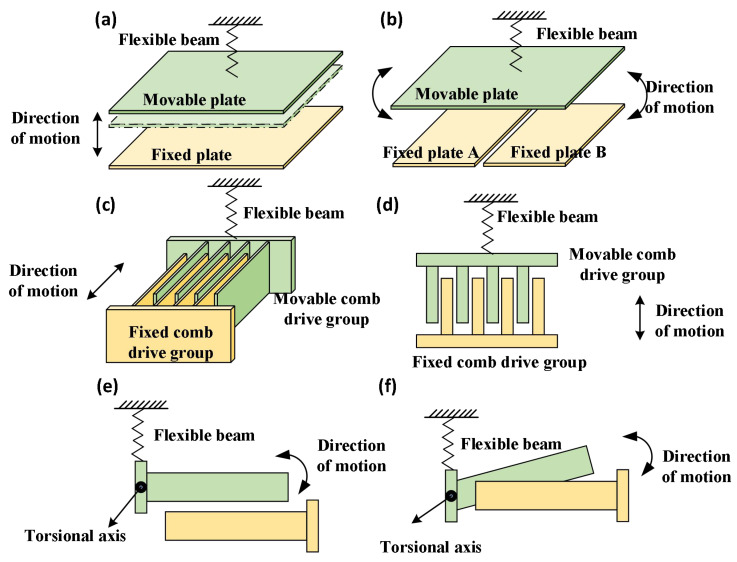
Schematic diagram of electrostatic-driven structure: (**a**) Flat Drive Structure (for Axial Motion); (**b**) Flat Drive Structure (for Torsional Motion); (**c**) Planar Comb-Drive Structure; (**d**) Planar Comb-Drive (Top View); (**e**) Offset Vertical Comb-Drive Structure; (**f**) Angled Vertical Comb-Drive Structure.

Electrostatic-driven structures primarily comprise two categories: flat-plate drives [29] and comb-drive mechanisms [30]. Schematic diagrams of the flat-plate drive are shown in Figure 1a,b, which consist of two sets of parallel plates: one fixed plate and one movable plate. The movable plate is connected to a fixed anchor point via a micro-elastic beam. Planar comb drive mechanisms comprise a set of fixed comb teeth anchored to the substrate and a set of movable comb teeth supported by elastic structures. These are arranged in an interlaced pattern to form the comb structure, as illustrated schematically in Figure 1c,d. Vertical comb drive mechanisms present two variants: staggered vertical comb drives comprise multiple pairs of movable and fixed comb teeth arranged with height differences in the vertical plane, as schematically illustrated in Figure 1e, while inclined vertical comb drives comprise multiple pairs of movable and fixed comb teeth with an initial angular offset, as schematically illustrated in Figure 1f. When a voltage is applied between the movable and fixed structures, the movable structure moves towards the fixed structure under electrostatic force. Upon removal of the drive voltage, the movable structure returns to its equilibrium position under the elastic restoring force of the micro-elastic beam.

Among the three types of electrostatic drive structures, the planar comb drive offers a relatively simple fabrication process and can deliver a sufficiently large deflection angle, making it the preferred choice for this design. In contrast, conventional planar drives suffer from an overly small deflection angle and pronounced electrostatic pull-in effects, while vertical comb drives, although capable of a larger deflection angle, entail more complex manufacturing steps.

**Figure 2:**
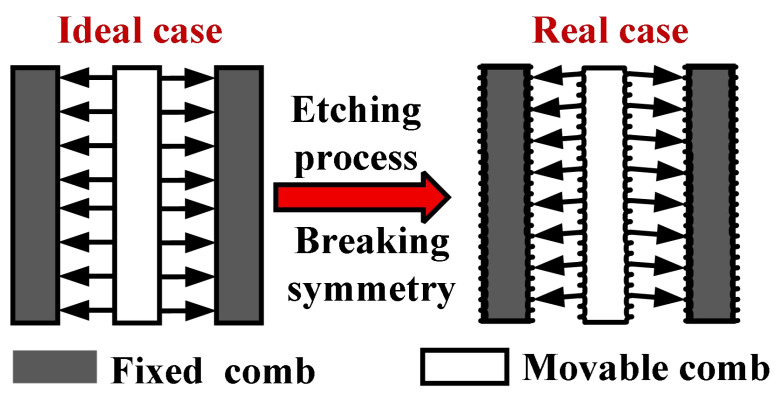
Comparison of ideal versus actual etching process outcomes.

Given the structural characteristics of planar comb teeth, they are suitable for achieving axial motion within the plane; however, this design requires out-of-plane torsional motion. During the etching step of device fabrication, process limitations introduce asymmetry into the comb tooth sidewalls, as illustrated in Figure 2. This asymmetry arises from the deep silicon etching process, which utilizes the Bosch process in the etching equipment. The essence of this process is a time-multiplexed reactive ion etching technique. It is not a continuous etching procedure but achieves highly anisotropic etching by rapidly and alternately executing two steps, namely, etching and passivation. Due to the cyclic nature of etching and passivation, periodic wavy undulations resembling scallop patterns form on the sidewalls. These patterns created on the sidewalls lead to asymmetry in the driving comb structure’s sidewalls, resulting in a slight torsional angle when a driving voltage is applied.

**Figure 3:**
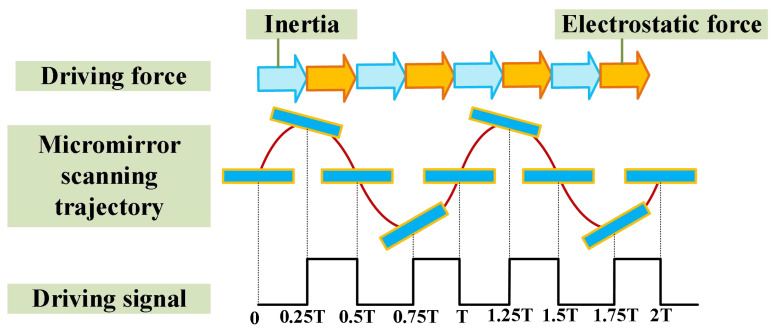
Principle diagram of micromirror operation outcomes.

The input voltage used to drive the torsional motion of the micromirror can take various waveforms, including sinusoidal, triangular, and square waves. A square wave with a 50% duty cycle provides the highest driving efficiency for the micromirror, assuming the driving frequency and peak-to-peak amplitude are the same across the three waveform types. Therefore, this paper adopts a 50% duty cycle square wave as the driving signal for the micromirror. Figure 3 illustrates the dynamic motion process of the micromirror.

At t=0, the micromirror surface is at its equilibrium position, with a scanning angle of 0 and maximum angular velocity. At this moment, all the energy of the micromirror is converted into kinetic energy. During 0<t<0.25T, the driving voltage is off and electrostatic force does no work. The micromirror deflects away from the equilibrium position due to inertia, converting its kinetic energy into the elastic potential energy of the torsional beam and energy dissipated by damping. At t=0.25T, the scanning angle of the micromirror reaches its maximum, with an angular velocity of 0. At this moment, all the energy of the micromirror is stored as the elastic potential energy of the torsional beam. During 0.25T<t<0.5T, the driving voltage is on and electrostatic force performs positive work. The micromirror begins to deflect back toward the equilibrium position under the electrostatic force. During this phase, the positive work done by the electrostatic force and the stored elastic potential energy are all converted into kinetic energy of the micromirror and energy dissipated by damping. At t=0.5T, the micromirror returns to its equilibrium position, with a scanning angle of 0 and maximum angular velocity. At this moment, all the energy of the micromirror is converted into kinetic energy. During 0.5T<t<0.75T, the driving voltage is off and electrostatic force does no work. The micromirror deflects away from the equilibrium position again. During 0.75T<t<T, the driving voltage is on and the micromirror deflects back toward the equilibrium position under the electrostatic force.

As shown in Figure 3 and the corresponding explanation, the micromirror completes one full torsional motion throughout the entire period from 0 to T, during which it experiences two square waves. Therefore, when the micromirror is in resonance, the frequency of the driving signal is twice the resonant frequency of the micromirror. Changing the driving voltage of the driving signal increases the torsional angle. Specifically, during testing, a square wave signal with an amplitude of 5 V and a driving frequency equal to twice the simulated resonant frequency is output by a signal generator. This signal is amplified by a voltage amplifier, and the driving signal frequency is adjusted until the observed light path on the screen reaches its maximum length. At this point, half of the driving frequency is the final resonant frequency of the fabricated micromirror.

### 2.2. Microscope Performance Parameters

**Figure 4:**
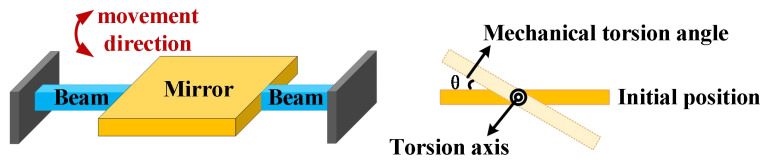
Schematic of one-dimensional torsional micromirror structure.

The torsional motion of MEMS mirrors in resonance can be regarded as torsional vibration. Figure 4 presents a simplified schematic of a one-dimensional torsional mirror structure. The dynamic equations are as follows [31]:

(1) $$I\ddot{\theta }+c\dot{\theta }+k\theta =T.$$

In the above equation, θ denotes the angular displacement varying with time, $\dot{\theta }$ represents the angular velocity, $\ddot{\theta }$ indicates the angular acceleration, T signifies the driving torque, c denotes the damping coefficient, and k denotes the damping stiffness.

When the micromirror undergoes torsional motion, the formula for calculating the elastic constant of the rectangular torsion axis is as follows [31]:

(2) $$K_{S}=\frac{2GK_{hb}hb^{3}}{l}.$$

In this formula, G denotes the shear modulus, h represents the half-shaft height of the torsion beam, b indicates the half-shaft width of the torsion beam, and L signifies the length of the torsion axis, where

(3) $$K_{hb}\left\{\begin{array}{c} 5.53-3.36\frac{h}{b}\left(1-\frac{b^{4}}{12h^{4}}\right),h>b\\ 5.53-3.36\frac{h}{b}\left(1-\frac{h^{4}}{12b^{4}}\right),h<b. \end{array}\right.\;$$

In MEMS micromirror structures, calculation of the rotational inertia requires consideration of the mirror’s structural configuration and mass distribution. Its rotational inertia may be expressed as [31]

(4) $$I_{m}=\int I^{2}dm=\int \rho \omega tl^{2}=\frac{1}{3}\rho \omega t\left(l_{1}^{3}-l_{0}^{3}\right),$$

where ω denotes width, ρ denotes density, t denotes thickness, and $l_{1}$ and $l_{0}$ respectively denote the distances from the target component to the distal and proximal ends of the torsion beam. When the micromirror operates in torsional resonance mode, its natural frequency may be expressed as follows [32]:

(5) $$f=\frac{\omega }{2\pi }=\frac{1}{2\pi }\sqrt{\frac{K_{S}}{I_{m}}}.$$

**Figure 5:**
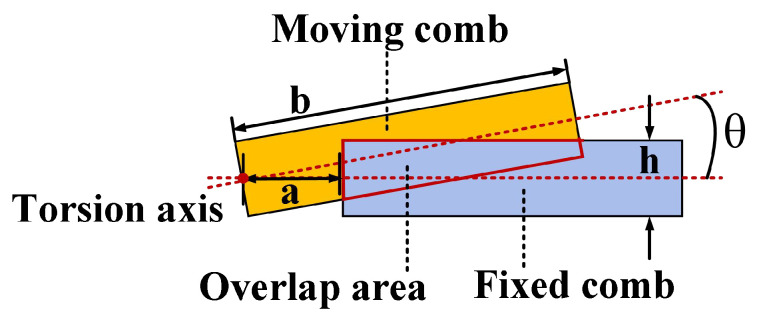
Comb drive capacitive torsion schematic.

Figure 5 illustrates the structural schematic of a pair of planar comb tooth capacitors undergoing torsion. The variation in capacitance between the comb teeth arises from changes in the overlapping area. The expression for the overlapping area of the comb tooth capacitors is given by Equation (6). As the torsion angle alters, the overlapping area changes, with its rate of change with angle expressed by Equation (7). Since alterations in the overlapping area cause capacitance to vary, the rate of change of capacitance with the angle is given by Equation (8). Consequently, the magnitude of the electrostatic torque generated by a pair of comb tooth capacitors is expressed by Equation (9). Calculating the number of comb tooth pairs yields the total electrostatic torque produced by the entire comb tooth capacitor array, as shown in Equation (10) [33].

(6) $$A\left(\theta \right)=\left\{\begin{array}{c} h\left(b-a\right)-\frac{1}{2}\left(b^{2}-a^{2}\right)\theta ,\left(0<\theta <\frac{h}{b}\right)\\ \frac{{\left(h-a\theta \right)}^{2}}{2\theta },\left(\frac{h}{b}<\theta <\frac{h}{a}\right) \end{array}\right.$$

(7) $$\frac{\partial A(\theta )}{\partial \theta }=\left\{\begin{array}{c} -\frac{1}{2}\left(b^{2}-a^{2}\right),\left(0<\theta <\frac{h}{b}\right)\\ -\frac{h^{2}}{2}+\frac{a^{2}}{2},\left(\frac{h}{b}<\theta <\frac{h}{a}\right) \end{array}\right.$$

(8) $$\frac{\partial C(\theta )}{\partial \theta }=\frac{\varepsilon }{g}\frac{\partial A(\theta )}{\partial \theta }=\left\{\begin{array}{c} \frac{\varepsilon }{g}\times \left[-\frac{1}{2}\left(b^{2}-a^{2}\right)\right],\left(0<\theta <\frac{h}{b}\right)\\ \frac{\varepsilon }{g}\times \left[-\frac{h^{2}}{2}+\frac{a^{2}}{2}\right],\left(\frac{h}{b}<\theta <\frac{h}{a}\right) \end{array}\right.$$

(9) $$m=\frac{\partial C(\theta )}{\partial \theta }V^{2}$$

(10) $$M=N\frac{\partial C(\theta )}{\partial \theta }V^{2}$$

In the above formula, a denotes the distance from the proximal end of the fixed comb tooth to the torsion axis, b denotes the distance from the distal end of the moving comb tooth to the torsion axis, g denotes the spacing between comb teeth, h denotes the thickness of the comb tooth, V denotes the drive voltage, N denotes the logarithm of the comb tooth, and ε denotes the air dielectric constant.

## 3. Device Structural Design and Performance Simulation

### 3.1. Device Structural Design

**Figure 6:**
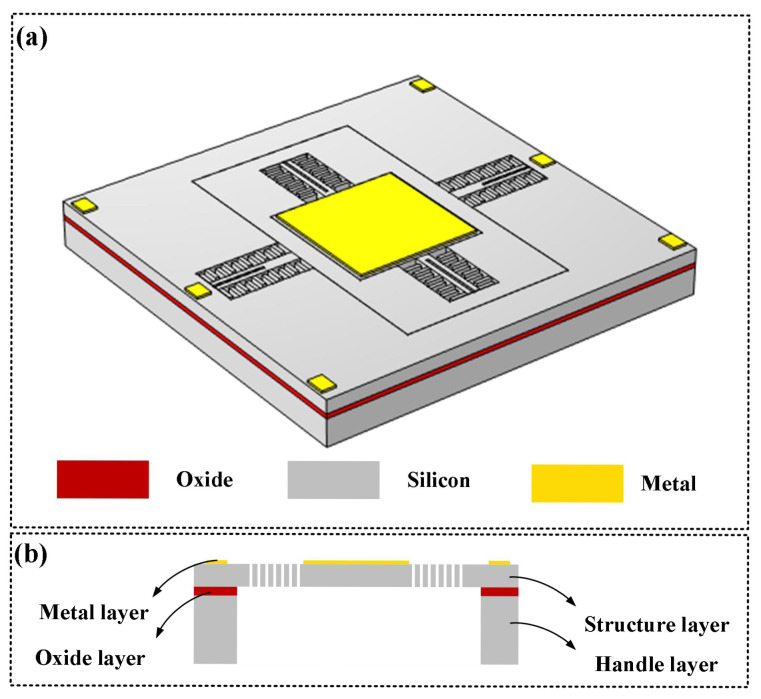
3D structural diagram of MEMS micromirror: (**a**) 3D diagram; (**b**) sectional drawing.

Figure 6a presents a schematic diagram of the three-dimensional structure designed herein, comprising four layers: a metallic layer, a silicon device layer, an insulating layer, and a silicon substrate layer. Figure 6b depicts its cross-sectional view. From the three-dimensional schematic, the structure is arranged from inner to outer as follows: a mirror surface, a fast-axis planar comb drive mechanism, a slow-axis torsional frame, a slow-axis planar comb drive mechanism, and an anchor region. The axis with the higher resonant frequency is generally termed the fast axis, while the axis with the lower resonant frequency is termed the slow axis.

**Figure 7:**
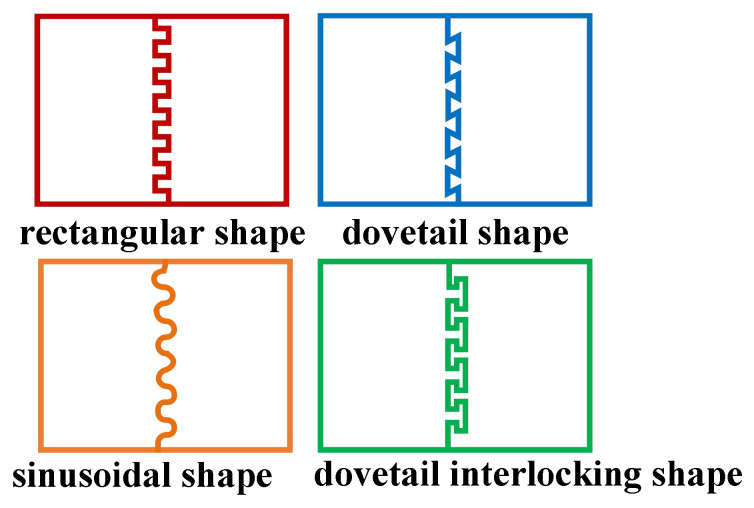
Schematic of isolation trench.

The driving potentials for MEMS micromirrors comprise three types: fast-axis drive voltage, slow-axis drive voltage, and ground terminal. The driving potential diagram corresponding to the device designed herein is illustrated in Figure 6. To achieve mechanical coupling and electrical isolation of the device, an isolation channel must be incorporated. Common shapes for isolation channels include rectangular, dovetail, sinusoidal, and dovetail interlocking. A schematic of these structures is presented in Figure 7. Considering the shear stress at the joint interface and the maximum normal stress, the dovetail interlocking configuration can withstand greater concentrated forces and bending moments compared to the other three structures. Consequently, this configuration offers the highest connection strength for isolation channel grooves. Therefore, this shape was adopted for the isolation channel in the present design.

**Figure 8:**
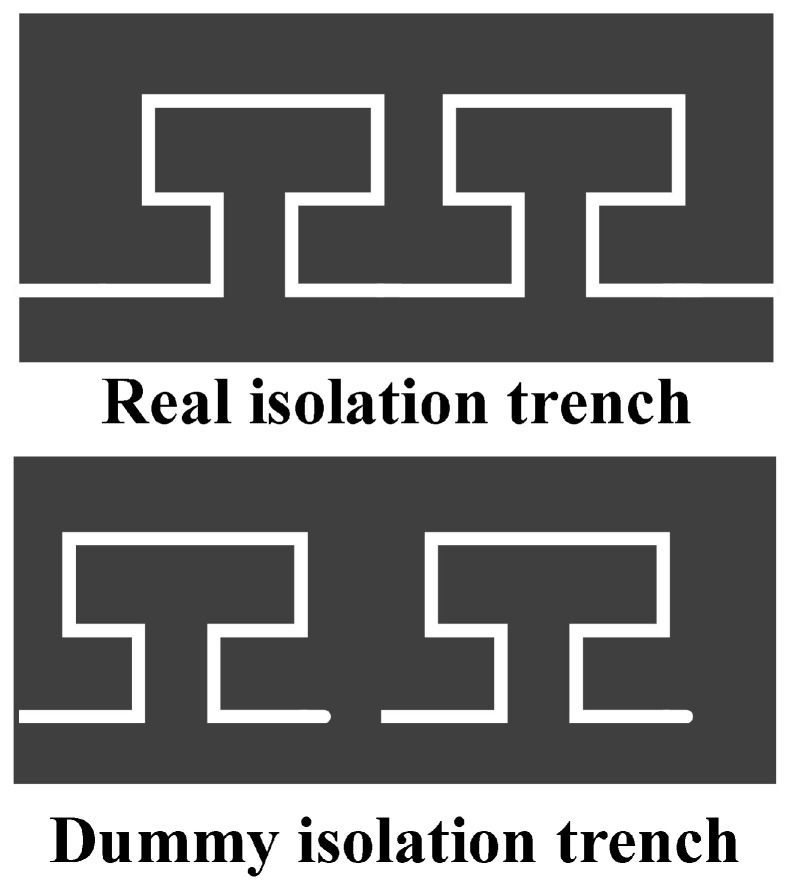
Schematic diagram of true/false channel structure.

**Figure 9:**
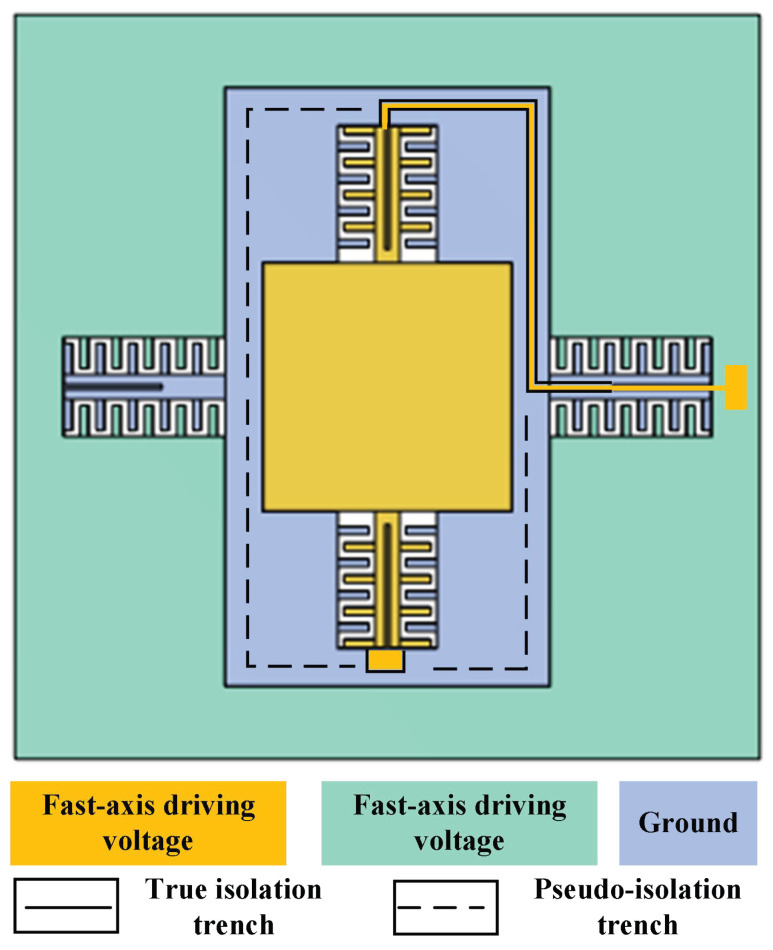
Potential distribution map of MEMS micromirror.

Introduction of isolation trenches enables both mechanical connection and electrical isolation to be achieved simultaneously. However, due to differences in the filling material of the isolation trenches compared to the rest of the device, an imbalance in the overall mass of the device occurs. This imbalance may ultimately lead to oscillation phenomena in the micromirror. To prevent this issue, dummy trenches are positioned symmetrically relative to the isolation trenches in order to achieve mass balance across the entire device. The schematic diagram of the true and dummy trench structure is shown in Figure 8, with their distribution within the overall device illustrated in Figure 9.

### 3.2. Performance Simulation of the Micromirror

We performed relevant performance simulations on the designed MEMS micromirror, primarily encompassing modal analysis, electrostatic force analysis, and harmonic response analysis, in order to validate the resonant frequencies and amplitude–voltage relationships of the MEMS micromirror’s fast axis and slow axis. Table 2 presents the relevant structural parameters of the MEMS micromirror.

**Figure 10:**
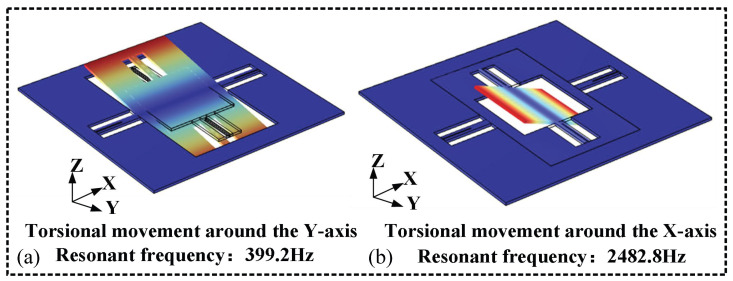
Modal analysis diagram of MEMS micromirror: (**a**) slow axis mode; (**b**) fast-axis mode.

Figure 10 presents the modal analysis diagram of the MEMS micromirror designed in this study. It is capable of rotational motion about the XY axes, with the resonant frequencies of the two motion modes exhibiting a significant difference in order to prevent coupling between axes.

To determine the torsional angle achievable by the micromirror under varying drive voltages, a simulation coupling the electrostatic and solid mechanics fields is required. However, the micromirror design utilises asymmetry introduced during fabrication along with resonance phenomena to achieve torsional motion, while the model established in finite element simulation software is entirely symmetrical. Thus, an indirect simulation approach is employed to study the micromirror’s amplitude–voltage relationship.

**Figure 11:**
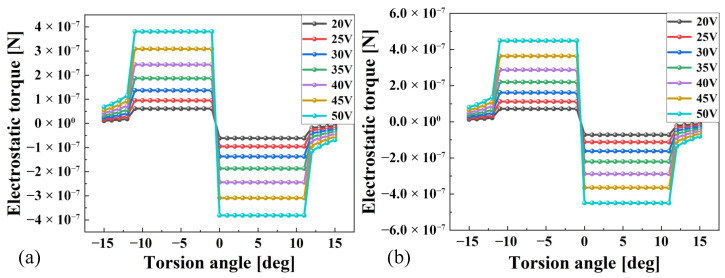
Electrostatic force simulation results for MEMS micromirror: (**a**) fast axis; (**b**) slow axis.

The core methodology of this simulation approach consists of constructing a model for a pair of comb tooth capacitors within a coupled electrostatic–solid mechanics multiphysics framework. By applying a voltage and rotating the movable comb teeth, the capacitance value corresponding to the twist angle at that drive voltage is obtained. Performing a parametric scan on the rotation angle of the movable comb teeth yields the capacitance values at different twist angles for a given drive voltage. By varying the driving voltage, the relationship between the twist angle and capacitance value at different voltages is established. Applying Equation (8) to the simulated capacitance results yields the rate of capacitance variation with angle. The electrostatic torque generated by this comb capacitor can be calculated using Equation (9). Combining the designed number of comb teeth with Equation (10) enables calculation of the electrostatic force magnitude produced by this drive structure under different drive voltages. The magnitudes of the electrostatic torques on the fast and slow shafts are shown in Figure 11a and Figure 11b, respectively.

**Figure 12:**
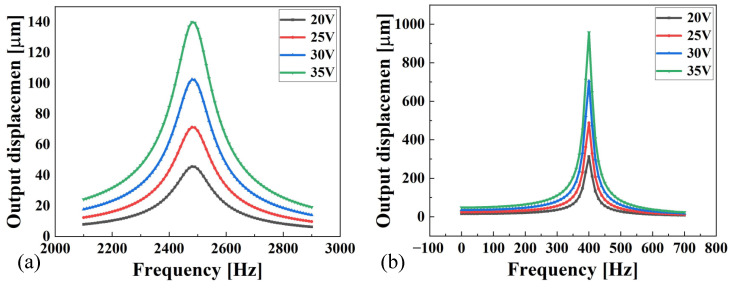
Frequency response simulation results for the MEMS micromirror: (**a**) fast axis; (**b**) slow axis.

**Figure 13:**
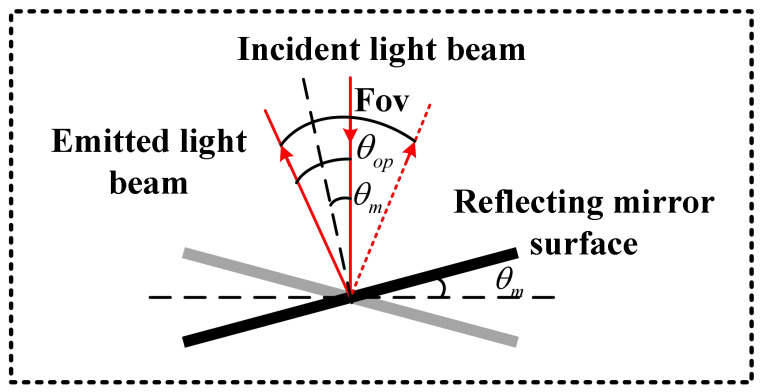
Parameter conversion relationships for the MEMS micromirror.

**Figure 14:**
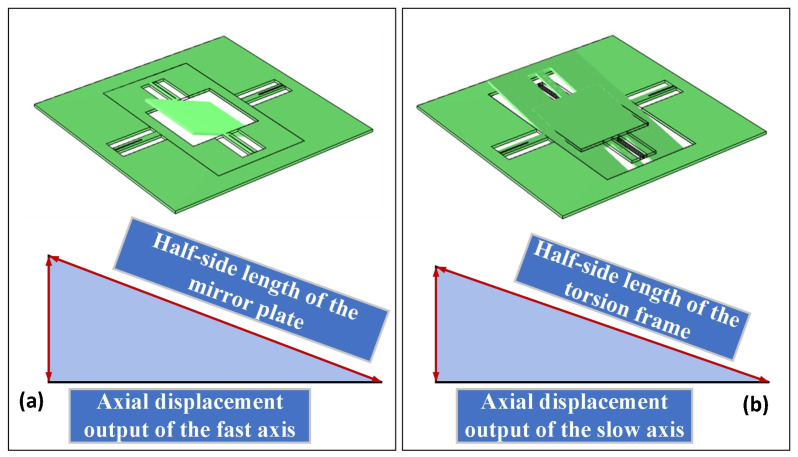
Specific conversion relationships for the MEMS Micromirror: (**a**) fast axis; (**b**) slow axis.

By obtaining the electrostatic torque generated by the drive structure under different drive voltages, this torque can be applied to the corresponding drive structure to determine the out-of-plane axial displacement produced during MEMS micromirror resonance at that voltage. Simulation results for the fast and slow axes are shown in Figure 12a and Figure 12b, respectively. Mirror performance is typically evaluated using the torsional angle parameter, necessitating conversion of the obtained axial displacement output. The conversion relationship is illustrated in Figure 13, where $\theta_{op}$ denotes the optical scanning angle, $\theta_{m}$ represents the mechanical scanning angle, and FOV indicates the field of view angle. Their relationship is expressed by Equation (11). The simulation results in Figure 11 are converted to derive the mirror’s amplitude–voltage correspondence. The specific conversion diagrams for the fast and slow axes are shown in Figure 14. Calculations are performed using the inverse sine function. For the fast axis, the half-length of the mirror surface and the simulated axial displacement output are utilised, as depicted in Figure 14a. For the slow axis, the half-length of the torsion frame and the axial displacement output are employed, as depicted in Figure 14b. The inverse sine function yields the magnitude of the mechanical twist angle. Doubling this value yields the magnitude of the optical twist angle. Conversion to the optical twist angle facilitates direct comparison with experimental results, since the measurements directly provide optical twist angles. The amplitude–voltage relationships for the fast and slow axes are presented in Table 3.

(11) $$FOV=4\theta_{m}=2\theta_{op}$$

## 4. Device Fabrication and Testing

### 4.1. Process Design and Fabrication of Devices

**Figure 15:**
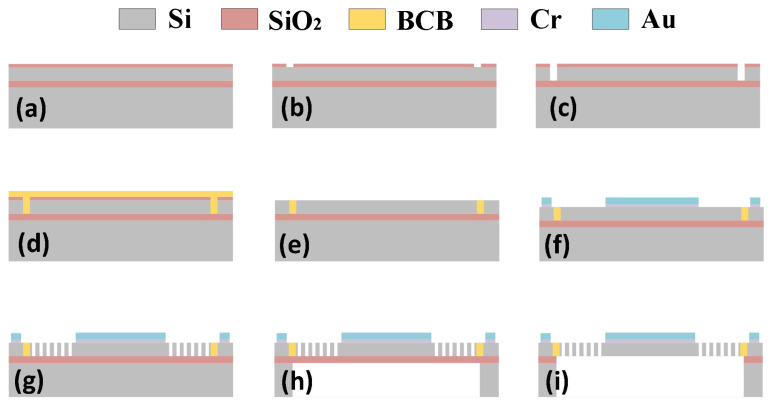
Process flow diagram for the MEMS micromirror: (**a**) silicon oxide growth; (**b**) etching of silicon oxide layer (isolation trench layer); (**c**) etching of silicon layer (isolation trench layer); (**d**) BCB filling; (**e**) mechanical polishing (CMP); (**f**) magnetron sputtering; (**g**) etching of silicon layer (structural layer); (**h**) etching of silicon layer (back cavity layer); (**i**) release.

This design employs an electrostatic-driven MEMS micromirror fabricated using bulk silicon processing techniques within MEMS manufacturing. A silicon-on-insulator (SOI) wafer was selected to realise the fabrication of the entire structure. The MEMS micromirror was progressively fabricated through a four-layer structure comprising the structural layer, isolation trench layer, metallisation layer, and back cavity layer. Figure 15 illustrates the process flow diagram for this project [34].

**Figure 16:**
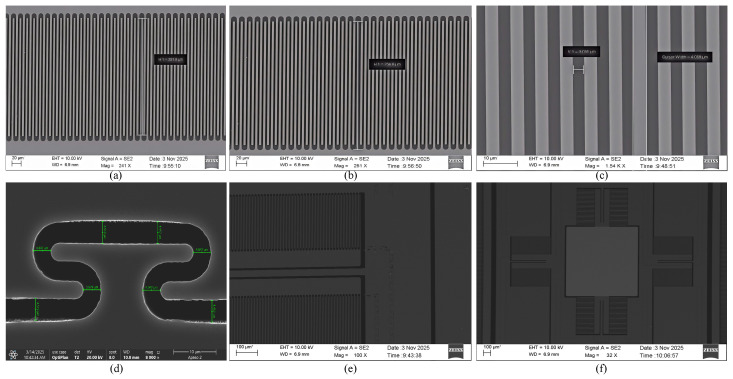
Local SEM images of the MEMS micromirror: (**a**) comb finger overlap length; (**b**) comb finger length; (**c**) comb finger gap width; (**d**) isolation trench; (**e**) partial view of comb fingers; (**f**) partial view of mirror.

First, a layer of silicon dioxide is grown upon the top silicon layer of the SOI wafer, as shown in Figure 15a. Subsequently, the isolation trench layer undergoes photolithography and etching; first, the silicon dioxide layer is patterned, as depicted in Figure 15b, then the isolation trench pattern is fabricated on the top silicon layer, as illustrated in Figure 15c. Next, the isolation material benzocyclobutene (BCB) is filled under vacuum suction conditions, as shown in Figure 15d. Following curing, surface BCB is removed; the majority is stripped via the grown silica layer and residual BCB is cleared by chemical mechanical polishing (CMP), as shown in Figure 15e. Mirror surfaces and pads are formed via magnetron sputtering, as depicted in Figure 15f. Photolithography and etching are performed on the top silicon layer to pattern the structural layer of the micromirror, as shown in Figure 15g. Photolithography and etching are subsequently applied to the back cavity layer, as depicted in Figure 15h. Finally, the SOI buried oxide layer is removed to release the mirror and movable structures, as illustrated in Figure 15i. Figure 16 presents local SEM images captured during the fabrication process.

**Figure 17:**
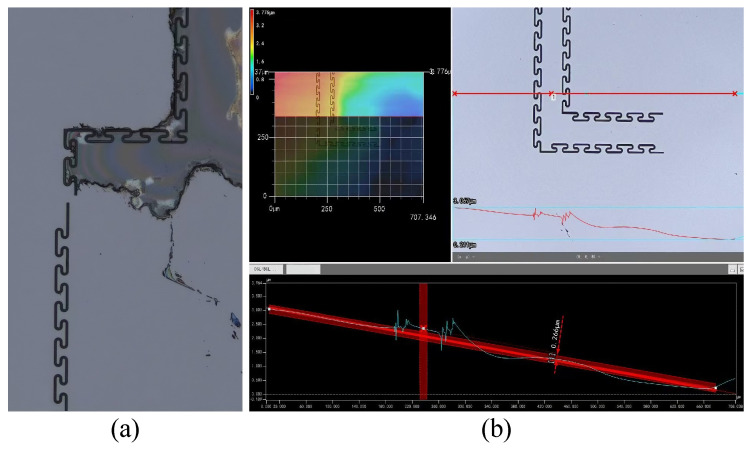
Comparison of BCB removal effects: (**a**) BCB removal effect without oxygen-doped silicon growth; (**b**) effect after oxygen-doped silicon growth.

During this processing step, the growth of oxygen-doped silicon enables a cleaner wafer surface during subsequent BCB removal. Figure 17a illustrates the BCB removal effect without oxygen-doped silicon growth, while Figure 17b shows the effect after oxygen-doped silicon growth. It is evident that BCB removal is more thorough after oxygen-doped silicon growth. Furthermore, this growth approach significantly reduces the BCB removal time. Without oxygen-doped silicon growth, CMP must be employed for BCB removal, which is time-consuming. With this growth approach most BCB is removed during oxygen–silicon stripping, followed by CMP to clear residual BCB, thereby saving substantial time.

Based on the above, growing and patterning a silicon oxide layer before filling can improve the cleanliness of the wafer after BCB removal as well as reduce the time cost. For this specific design, the growth parameters are as follows: using Sentech Si 500D equipment in the Micro-Nano Fabrication Center of Beijing Institute of Technology Chongqing Institute of Microelectronics (The machine was manufactured in Berlin, Germany by SENTECH Instruments GmbH.), silicon dioxide is deposited on the surface of the SOI device layer; the deposited silicon oxide thickness is 200 nm, the deposition temperature is 300 °C, the power is 500 W, and the deposition time is 918 s. During the removal process, a silicon dioxide polishing slurry is first used to remove the silicon dioxide layer. Once a significant amount of BCB is observed to have been removed from the surface, the process is switched to a silicon polishing slurry to remove the remaining BCB residue.

**Figure 18:**
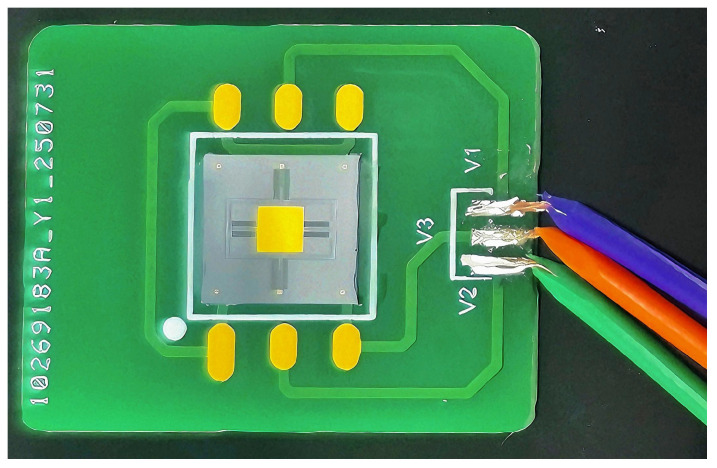
Connection diagram of micromirror to circuit interface board.

The prepared device was then connected to the circuit adapter board via gold wire bonding to facilitate subsequent testing of the micromirror. Figure 18 shows the overall configuration of the device connected to the circuit adapter board.

### 4.2. Performance Testing of Components

**Figure 19:**
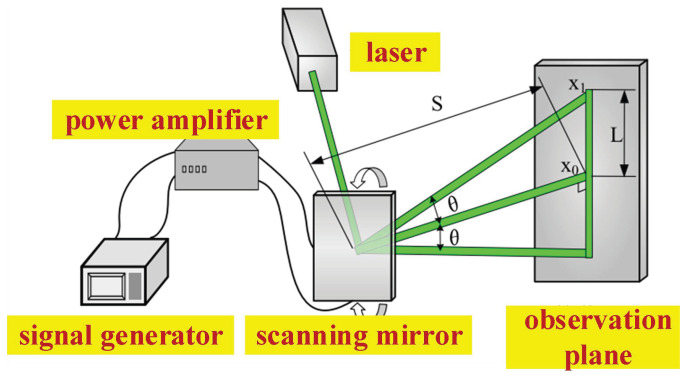
Principle diagram of laser triangulation method.

**Figure 20:**
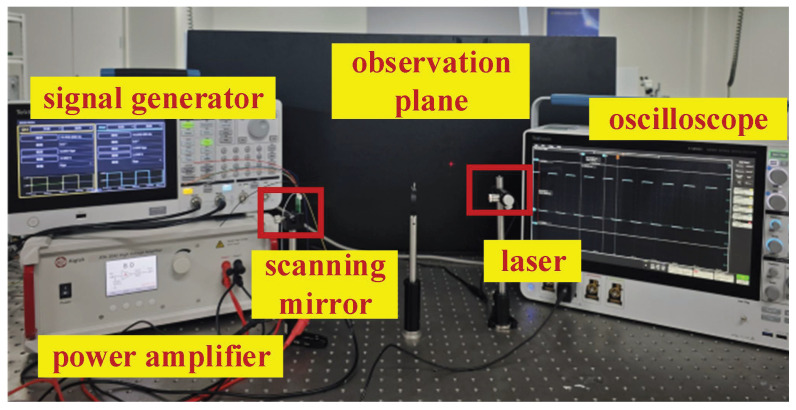
Optical test platform.

Performance testing of the fabricated electrostatic micromirror was conducted using laser triangulation [35]. The testing principle is illustrated in Figure 19, wherein the distance from the laser to the mirror surface and the length of the light ray on the observation plane are measured. The optical twist angle of the micromirror is then determined according to Equation (12). The constructed optical testing platform is depicted in Figure 20.

(12) $$\theta =\arctan\frac{L}{2S}$$

The principle of measuring the torsional angle of the micromirror using the experimental setup diagram shown in Figure 20 is as follows. After setting up the experimental platform, a red helium–neon laser is used to emit a laser beam, which irradiates the surface of the MEMS micromirror. The laser reflects off the surface of the MEMS micromirror and produces a light spot on the observation screen. By applying a corresponding driving voltage signal to the MEMS micromirror through a signal generator and power amplifier, the MEMS micromirror undergoes torsional motion. As the MEMS micromirror twists, the light spot on the observation screen moves rapidly, forming a line segment. By measuring the length of this laser line segment on the observation screen and the distance from the mirror surface to the observation screen using a ruler, the torsional angle of the MEMS micromirror at that moment can be calculated according to Equation (12).

**Figure 21:**
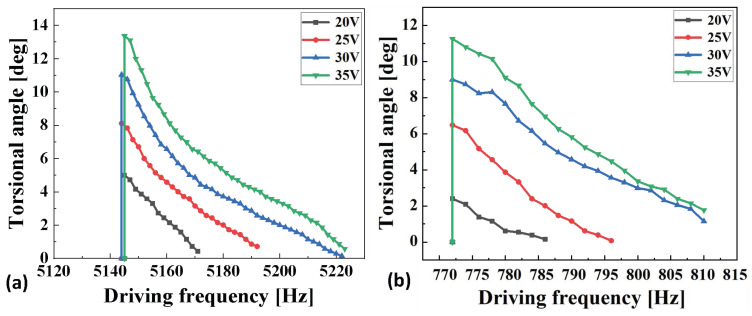
Frequency response test results for MEMS micromirror: (**a**) fast axis; (**b**) slow axis.

**Figure 22:**
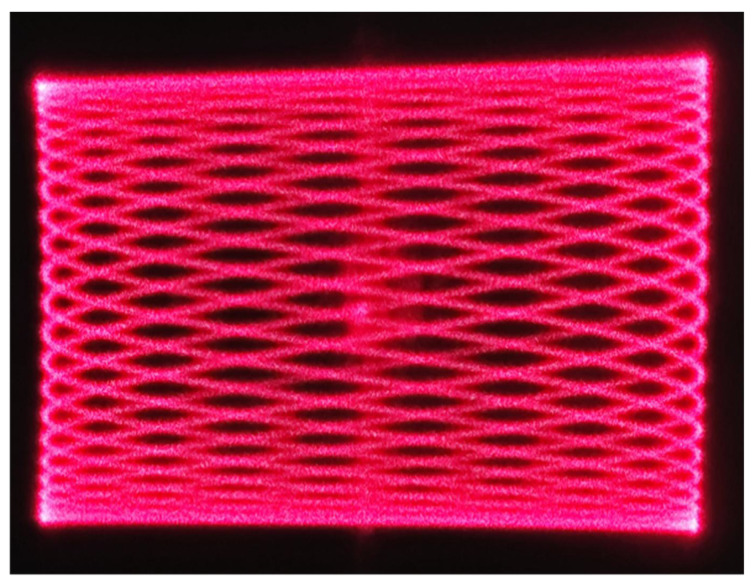
Lissajous scan patterns.

The twist angle of the micromirror is dependent upon both the drive voltage and the drive frequency. By maintaining a fixed drive voltage and adjusting the drive frequency, the twist angle at different frequencies can be obtained. This test constitutes the frequency response test for the micromirror. Test results are presented in Figure 21. Figure 21a displays the frequency response test results for the fast axis, while Figure 21b shows the frequency response curve for the slow axis. As both torsion axes operate in resonant mode, their scan patterns exhibit Lissajous figures, as illustrated in Figure 22.

As shown in Figure 21, at a drive voltage of 35 V, the fast and slow axes attain their maximum twist angles at drive frequencies of 5140 Hz and 770 Hz, respectively. This indicates that the MEMS mirror is in a resonant state at these frequencies. The resonant frequency for this particular mode of motion is half the drive frequency. Simple calculations reveal that the resonant frequencies for the fast and slow axes are 2570 Hz and 385 Hz, respectively, differing from the simulation results by 88 Hz and 14 Hz. Comparison reveals that the simulation results are broadly consistent with the test results.

Subsequently, the twist angle of the micromirror in its resonant state was tested by altering the drive voltage. This constitutes the amplitude–voltage test for the micromirror. Table 4 presents the results of this amplitude voltage test alongside a comparison with the simulation results. The test findings demonstrate that the results for both the fast axis and slow axis are largely consistent with the simulation outcomes.

The error between simulation and experiment was calculated according to the experimental test results. The frequency response test results reveal the actual resonant frequencies of the fast and slow axes. The difference between the simulated and experimental results for the resonant frequency of the fast axis is approximately 3.5%, while the difference for the slow axis is also around 3.5%. From the amplitude–voltage test results of the fast and slow axes, the actual torsion angles under different driving voltages can be obtained. The average error between the torsion angles of the fast axis under various driving voltages and the simulation results is 6.075%, whereas the average error for the slow axis is 14.025%. The possible reasons for these errors may include the following:

Process Non-Idealities: Due to manufacturing tolerances, there are discrepancies between the actual fabricated device and the ideal simulation model. Deviations in structural parameters directly affect mechanical stiffness and cause minor changes in capacitance, thereby influencing the resonant frequency and amplitude–voltage relationship and causing deviations from the simulation results.

Testing Errors: In addition to process-related factors, errors may also arise during testing. For example, environmental vibrations during frequency response testing could affect the measured resonant frequency. In amplitude–voltage testing, the nonlinearity of the voltage amplifier may introduce additional errors.

## 5. Conclusions

This study designed and fabricated an electrostatic-driven dual-axis MEMS micromirror capable of generating Lissajous scan patterns. Both axes employ planar comb drive structures, significantly simplifying fabrication complexity. During processing, oxygen–silicon growth is utilised to optimise BCB removal efficiency. The process is described sequentially: structural design, performance simulation, device fabrication, and performance testing. The fabricated MEMS micromirror exhibits a fast-axis resonance frequency of 2570 Hz and a slow-axis resonance frequency of 385 Hz, differing from the simulation results by 88 Hz and 14 Hz, respectively. The amplitude voltage test results for both the fast and slow axes also closely match the simulations, validating the feasibility of the indirect simulation approach and offering a methodology for simulating planar comb drive torsional motion performance. At 35 V, the fabricated micromirror achieves an optical twist angle of 13.36° along the fast axis and 11.24° along the slow axis, enabling a field of view of approximately 26°×22°. This design does not involve significant structural innovations, instead focusing on providing an indirect simulation analysis method and optimizing the process flow. These efforts effectively enhance wafer cleanliness and reduce time costs. Future research could explore greater innovations in structural aspects, such as modifying the shape of the torsion beam to enable larger resonant frequencies and torsion angles, which would facilitate application in fields such as projection displays based on Lissajous scanning patterns.

## Figures and Tables

**Table 1:** Comparison of characteristics of different drive methods for micromirrors.

Drive Method	Drive Voltage	Drive Force	Frequency	Range
Electrostatic Drive	High	Low	High	Low
Piezoelectric Drive	High	High	High	Low
Electromagnetic Drive	Low	High	High	High
Electrothermal Drive	Low	Medium	Low	High

**Table 2:** MEMS micromirror structural parameters.

Framework	Parameters
Mirror size	2 mm
Frame size	6 × 6 mm^2^
Comb length	250 µm
Comb width	4 µm
Clearance of the comb teeth	3 µm
Structure thickness	70 µm
Torsion beam length (Fast axis)	1000 µm
Torsion beam width (Fast axis)	30 µm
Torsion beam length (Slow axis)	800 µm
Torsion beam width (Slow axis)	20 µm
Number of combs (Fast axis)	78 × 4
Number of combs (Slow axis)	92 × 4

**Table 3:** Simulation results for the amplitude–pressure relationship of the MEMS micromirror.

Shaft	Voltage/V	Axial Displacement Output/µm	Torsional Angle/deg
Fast axis	20	46	5.273
	25	71	8.142
	30	100	11.478
	35	139	15.979
Slow axis	20	311	3.692
	25	487	5.793
	30	705	8.413
	35	957	11.004

**Table 4:** MEMS micromirror amplitude–voltage relationship test.

Shaft	Voltage/V	Test Data/deg	Simulation Data/deg
Fast axis	20	5.064	5.273
	25	8.110	8.142
	30	11.034	11.478
	35	13.360	15.979
Slow axis	20	2.398	3.692
	25	6.476	5.793
	30	8.994	8.413
	35	11.248	11.004

## Data Availability

The data used to support the findings of this study are available from the corresponding author upon request.
